# Pre-emptive hypothermia during resuscitated porcine hemorrhagic shock

**DOI:** 10.1186/cc10870

**Published:** 2012-03-20

**Authors:** J Matallo, W Stahl, M Gröger, A Seifritz, O Mccook, M Georgieff, P Asfar, M Matejovic, E Calzia, P Radermacher, F Simon

**Affiliations:** 1 University Medical School, UIm, Germany; 2 University Hospital, Angers, France; 3 Charles University, Plzeñ, Czech Republic

## Introduction

The role of hypothermia in hemorrhagic shock is still a matter of debate [1]. Therefore, we studied the effects of deliberate, pre-emptive hypothermia on hemodynamics and organ function during long-term porcine hemorrhage and resuscitation.

## Methods

Anesthetized and instrumented pigs were randomly assigned to 32°C (*n *= 7), 35°C (*n *= 7), and 38°C (*n *= 6) of core temperature and subjected to 4 hours of hemorrhage (removal of 40% of the calculated blood volume, additional removal/retransfusion of blood to maintain mean arterial pressure (MAP) = 30 mmHg). After 12 hours of reperfusion comprising retransfusion of shed blood, colloid fluid resuscitation and noradrenaline to keep MAP at pre-shock levels, animals were rewarmed to 38°C. Data (median, quartiles) were obtained before and at the end of the shock phase as well as at 12 and 22 hours of resuscitation, intergroup differences were analyzed using a Kruskal-Wallis ANOVA on ranks.

## Results

Fluid balance and noradrenaline requirements did differ between groups. At 12 hours of reperfusion - that is, immediately before rewarming - the 32°C group showed the lowest blood levels of creatinine (*P *= 0.026), troponin I (*P *= 0.053), the thrombin-antithrombin complexes (*P *= 0.012), and von Willebrand factor (*P *= 0.012). At the end of the experiment - that is, after rewarming - all these intergroup differences had disappeared, but the 32°C group presented with arterial hypotension (*P *= 0.039), the most severe visceral organ acidosis (portal and hepatic venous base excess: *P *= 0.044, *P *= 0.022, respectively), and the highest TNFα blood levels (*P *= 0.030).

## Conclusion

Deliberate, pre-emptive moderate hypothermia slowed but did not protect against hemorrhagic shock and resuscitation-induced organ dysfunction, possibly due to a delayed but not attenuated inflammatory response.
